# Biomarker identification associated with M2 tumor-associated macrophage infiltration in glioblastoma

**DOI:** 10.3389/fneur.2025.1545608

**Published:** 2025-05-14

**Authors:** Xue-yuan Li, Zhi-yun Yu, Hong-jiang Li, Dong-ming Yan, Chao Yang, Xian-zhi Liu

**Affiliations:** Department of Neurosurgery, The First Affiliated Hospital of Zhengzhou University, Zhengzhou, China

**Keywords:** glioblastoma, tumor-associated macrophage, prognostic signature, immune landscape, single cell

## Abstract

**Purpose:**

M2 phenotype tumor-associated macrophages (TAMs) can promote tumor growth, invasion, chemotherapy resistance and so on, leading to malignant progression. The aim of this study was to identify novel prognostic profiles in glioblastoma (GBM) by integrating single-cell RNA sequencing (scRNA-seq) with bulk RNA-seq.

**Methods:**

We identified M2-associated genes by intersecting TAM marker genes derived from scRNA-seq with macrophage module genes from WGCNA RNA-seq data. Prognostic M2 TAM-related genes were determined using univariate Cox and LASSO regression analyses. In the following steps, prognostic characteristics, risk groups, and external validation were constructed and validated. The immune landscape of patients with GBM was examined by evaluating immune cells, functions, evasion scores, and checkpoint genes.

**Results:**

Analysis of scRNA-seq and bulk-seq data revealed 107 genes linked to M2 TAMs. Using univariate Cox and LASSO regression, 16 genes were identified as prognostic for GBM, leading to the creation and validation of a prognostic signature for GBM survival prediction.

**Conclusion:**

Our findings reveal the immune landscape of GBM and enhance understanding of the molecular mechanisms associated with M2 TAMs.

## Introduction

Glioblastoma (GBM) is the most prevalent and aggressive primary malignant brain tumor in the central nervous system of adults (1). Despite aggressive treatment, the median survival time for patients with GBM is 10–14 months (2). Therefore, identifying “features” is crucial for predicting prognosis in patients with GBM.

The tumor microenvironment (TME), predominantly composed of immune cells, fibroblasts, and tumor cells, plays a role in the suboptimal treatment of gliomas (3). Tumor-associated macrophages (TAMs), predominantly found in the glioma TME, exhibit characteristics of alternatively activated (M2) macrophages and significantly contribute to tumor progression. M2 TAMs engage with various immune cells in the TME, diminishing anti-tumor immune cell populations and facilitating tumorigenesis (4). Consequently, it is critical to investigate how M2 TAMs affect the course of gliomas and develop a predictive signature associated with them.

In this study, we identified 16 M2 TAM-related genes through integrated analysis of single-cell and RNA-sequencing data, thereby constructing and validating prognostic features that predict prognosis in patients with GBM. The prognostic features were closely related to tumor immunoinfiltration. To better understand the immune environment in patients with GBM, we examined immunological differences between high-risk and low-risk groups.

## Materials and methods

### Data collection and analysis

Single-cell RNA sequencing (GSE147275) and bulk RNA-seq (GSE4290) data, along with clinical information for glioma were acquired from the Gene Expression Omnibus (GEO) database. Normalized RSEM gene-level RNA-seq data and associated clinical information from Rembrandt were obtained from Gliovis.^1^ The mRNA expression and clinical data for CGGA325 were downloaded from the Chinese Glioma Genome Atlas (CGGA).^2^ The “Seurat” R package was utilized for scRNA-seq data processing and analysis, filtering out cells with <20% mitochondrial gene content and >200 unique genes (5). This data is then applied to identify highly variable genes within cells. Principal component analysis identified significant principal components (PCs) of highly variable genes. Cell clustering was performed using t-distributed stochastic neighbor embedding (t-SNE) on the top 20 PCs. The “SingleR” package (6) was used for cell type annotation, whereas marker genes for cell clusters were identified using the “FindAllMarkers” function.

### M2 macrophage infiltration and M2 TAM-related genes

The relative content of M2 macrophages in the glioma samples was calculated using CIBERSORTx (7). Use the “survminer” package to determine the best cutoff value for distinguishing between high and low M2 macrophage content groups. The Survival of the low-and high-M2 macrophage groups was analyzed using the “Survival” R package.

The “WGCNA” R package (8) was used to perform weighted gene coexpression network analysis on TCGA glioma expression data, aiming to identify genes associated with M2 TAMs. The samples were clustered to assess their overall correlation and identify outliers. The picksoftthreshold function was utilized to compute the ideal softpower (9). Seven modules were produced when the minimum number of genes was 50. Modules relevant to M2 macrophage content were identified via correlation analysis between modules and traits. To identify M2 TAM-related genes, we intersected TAM marker genes from scRNA-seq analysis with M2 macrophage module genes from WGCNA.

### Development and validation of a prognostic signature associated with M2 TAMs

M2 TAM-related genes for prognostic feature construction were identified using univariate Cox and LASSO regression analyses. Patients with glioma were divided into low-and high-risk groups using the optimal risk score cutoff, and their survival differences were evaluated using Kaplan–Meier survival curves. The performance of the prognostic signature was assessed using ROC curves with the “survivalROC” package (10). The R package “GSVA” (11) was used to conduct gene set variation analysis to evaluate the potential functions of the signature genes. Pathways with significant enrichment were identified based on *p* < 0.05 and a false discovery rate <0.25 (12). Nomograms were created using the “rms” package, incorporating both the prognostic signature and clinical features, before evaluating using calibration curves.

### Cell culture

THP-1 cells were maintained in RPMI-1640 medium (Thermo Fisher Scientific, United States) supplemented with 10% fetal bovine serum (FBS) and were subsequently induced to differentiate into macrophages using 100 ng/mL Phorbol 12-myristate 13-acetate (PMA) (Sigma-Aldrich, United States). Macrophage M2 polarization was achieved through incubation with 20 ng/mL of interleukin-4 (IL-4; R&D Systems, catalog #204-IL) and 20 ng/mL of interleukin-13 (IL-13; R&D Systems, catalog #213-ILB).

### Real-time PCR

A total of 6 × 10^3^ THP1 cells were seeded into 6-well plates and subsequently treated with pharmacological agents for a duration of 24 h. Following treatment, the cells were washed once with phosphate-buffered saline (PBS), and total RNA was extracted utilizing the TRIzol reagent in accordance with the manufacturer’s instructions (Invitrogen). Subsequently, 800 ng of RNA was reverse transcribed into complementary DNA (cDNA) and subjected to amplification via real-time polymerase chain reaction (PCR) using a Bio-Rad system. The expression levels were normalized to the internal control gene, glyceraldehyde 3-phosphate dehydrogenase (GAPDH), for each replicate. The sequences of the primers employed are provided in the Supplementary Table S4.

### Immunofluorescence staining

For the immunofluorescence assay, human glioblastoma tumor tissues were initially fixed with 4% paraformaldehyde (PFA), followed by dehydration using 20 and 30% sucrose solutions. The samples were then embedded in an optimal cutting temperature compound (Sakura, CA, United States), rapidly frozen at −80°C, and sectioned into 8 μm coronal slices. Subsequently, the frozen sections were fixed again with 4% PFA for 30 min, blocked with 5% bovine serum albumin (BSA) for 1 h, and incubated with primary antibodies. Detection of the primary antibodies was achieved using Alexa Fluor™ 488-or 594-conjugated secondary antibodies (Life Technologies, Grand Island, United States). Confocal imaging was performed using the FluoView 1200 system (Olympus, Tokyo, Japan), ensuring consistent confocal scanning parameters across all samples. Image processing was minimal to preserve data integrity. The primary antibodies were used: anti-AIF1 (1:200, GTX632426, GeneTex), anti-ALOX5AP (1:200, orb101167, biorbyt), anti-CYBA (1:100, GTX133970, GeneTex), anti-FCER1G (1:200, GTX108487, GeneTex), anti-FCGR2A/CD32 (1:200, AF1330, R&Dsystems), anti-FPR1 (1:200, ABIN615853, antibodies-online), anti-MSR1 (1:200, orb1606994, biorbyt), Anti-NRAMP1/SLC11A1 (1:200, orb546332, biorbyt).

### Statistical analyses

**Figure 1:**
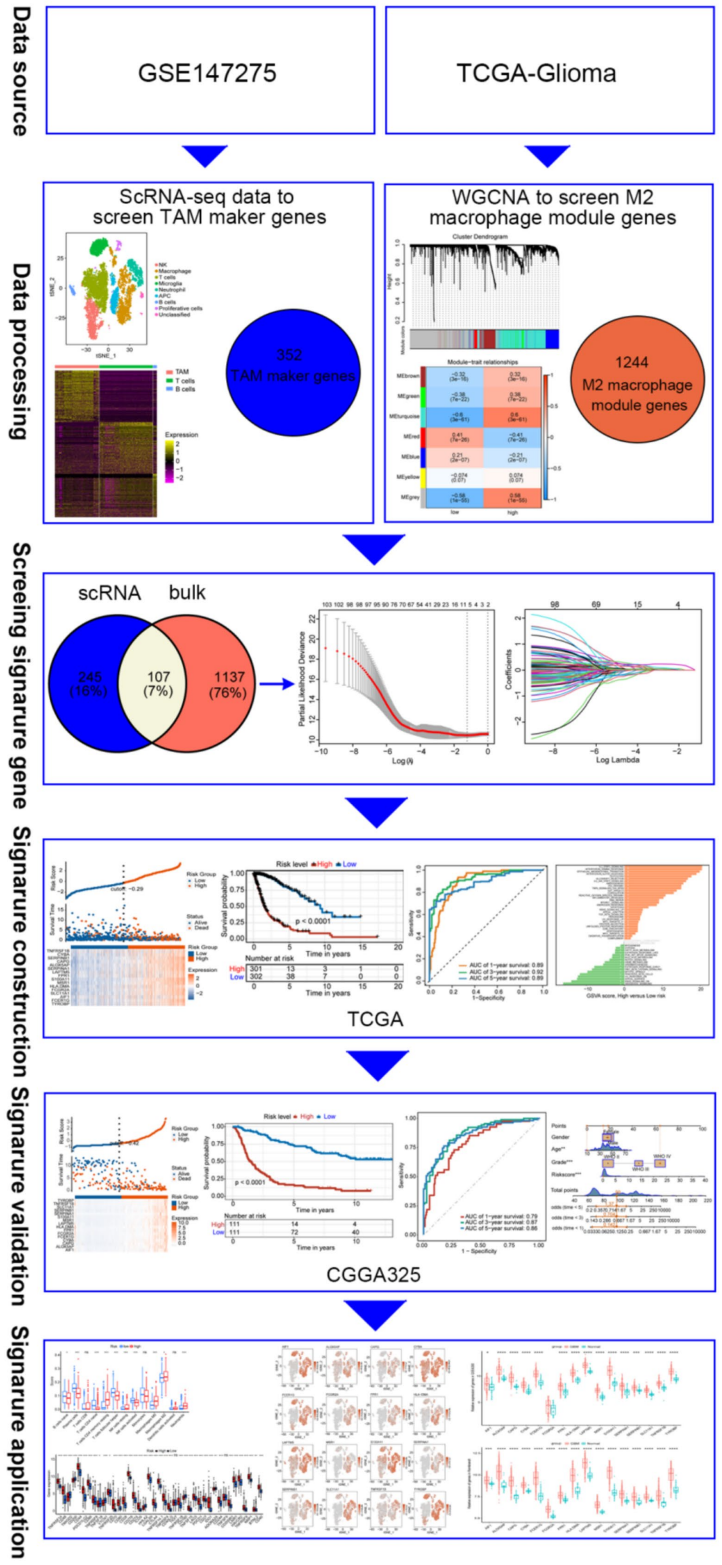
Diagram illustrating the data processing and analysis workflow.

Data were analyzed using R software (version 4.2.2). Survival analysis was performed using Kaplan–Meier. The *t-*test was used to validate differences between the two groups. A *p-*value <0.05 was considered statistically significant (see Figure 1).

## Results

### Identification of M2 macrophage-associated genes in GBM using WGCNA

**Figure 2:**
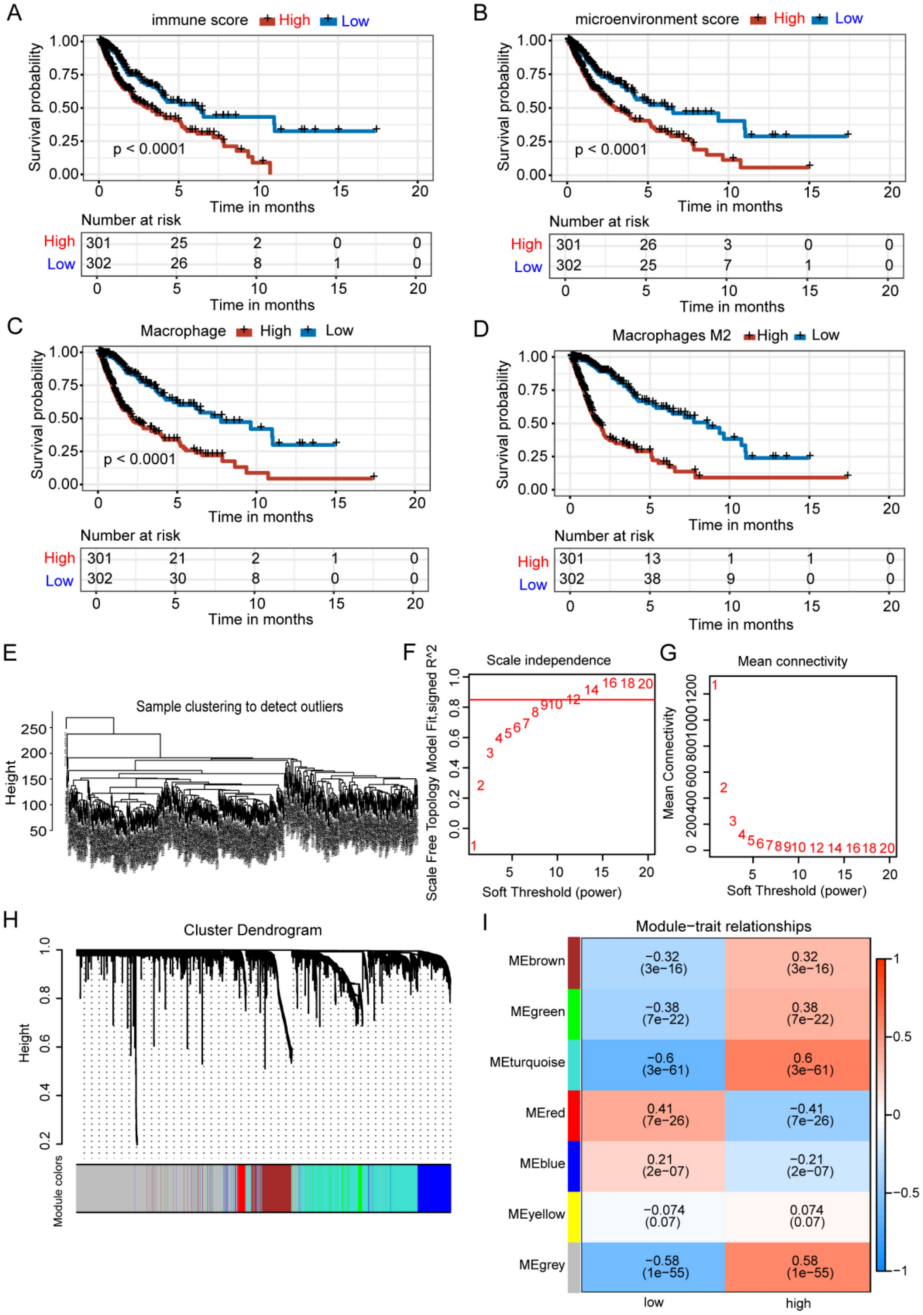
Identification of M2 TAM-associated genes in glioma using WGCNA. **(A-D)** Groups with elevated immune and microenvironment scores and higher levels of macrophages and M2 macrophages exhibited significantly poorer prognoses. **(E)** Samples were clustered without identifying any outliers. **(F-G)** The WGCNA package determined a soft threshold power of 9. **(H)** Correlation analysis identified seven non-gray modules associated with traits. **(I)** The turquoise module was identified as the most relevant module for M2 macrophages.

The role of the tumor immune microenvironment in glioma is being increasingly recognized. We divided TCGA glioma patients into high and low immune score groups to explore the relationship between tumor immune microenvironment and glioma prognosis Figure 1. Patients with glioma with low immune scores exhibited extended survival (Figure 2A). Patients with glioma with a high number of macrophages, particularly M2 macrophages, exhibited reduced survival rates, highlighting the crucial role of M2 macrophages in glioma prognosis (Figures 2B–D). The prognosis did not differ significantly between groups with varying levels of B cells, CD8^+^ T cells, NK cells, plasmacytoid dendritic cells, CD4^+^ memory T cells, CD4^+^ naive T cells, and CD4^+^ Th1 T cells. WGCNA was employed to identify genes associated with M2 macrophages in glioma. The results indicated no outliers in TCGA database (Figure 2E). The optimal soft-threshold power was determined to be 9 (Figures 2F,G), leading to the identification of seven modules (Figures 2H,I). The turquoise module exhibited the strongest correlation with high-M2 macrophage counts (*r* = 0.6, *p* < 0.0001) (Figure 2I).

### Screening for M2-TAM-related genes using scRNA-seq data

**Figure 3:**
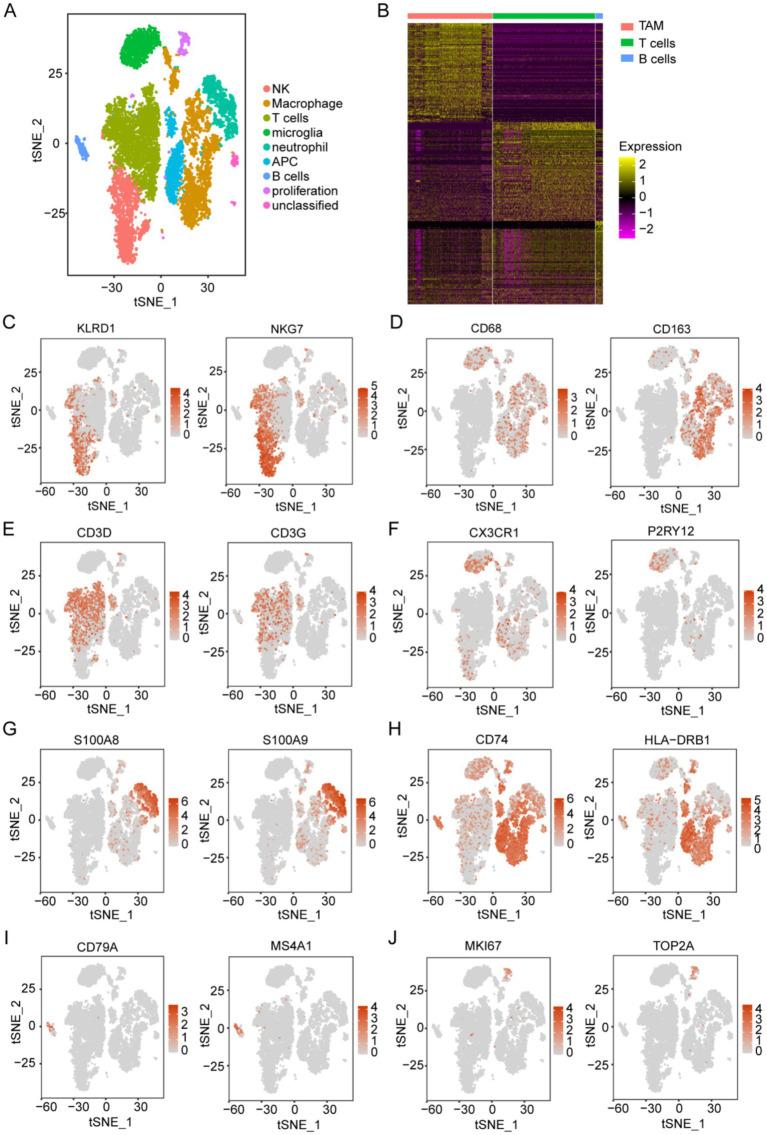
Acquisition of TAM marker genes using scRNA-seq data. **(A)** Analysis of immune components using t-SNE from 15 patients with glioma, based on the GSE147275 database, identified nine main clusters: NK cells, macrophages, T cells, microglia, neutrophils, APCs, B cells, proliferative cells, and unclassified cells. **(B)** The heatmap displays marker genes that exhibit differential expression in immune cells. **(C–J)** t-SNE of immune constituents showed representative markers for the following populations: **(C)** NK cells (KLRD1 and NKG7), **(D)** macrophages (CD68 and CD163), **(E)** T cells (CD3D and CD3G), **(F)** microglia (P2RY12 and CX3CR1), **(G)** neutrophils (S100A8 and S100A9), **(H)** APCs (CD74 and HLA-DRB1), **(I)** B cells (CD79A and MS4A1), and **(J)** proliferative cells (MKI67 and TOP2A).

For scRNA-seq analysis, 18,575 genes within 11,165 cells were identified. The Vlnplots illustrate the nFeature, nCount, and percent.mt per cell (Supplementary Figure S2A). The nCount showed a positive correlation with nFeature (correlation = 0.94) (Supplementary Figure S2B). The scatter diagram shows the top 2,000 variable genes, which are highlighted in red (Supplementary Figure S2C). Twenty PCs with high heterogeneity were selected for the t-SNE analyses (Supplementary Figures S2D,E). The t-SNE (Figure 3A) of immune constituents from 15 patients with glioma showed nine PCs: NK (Figure 3C), macrophage (Figure 3D), T cells (Figure 3E), microglia (Figure 3F), neutrophils (Figure 3G), APCs (Figure 3H), B cells (Figure 3I), proliferative cells (Figure 3J), and unclassified cells. We focused on the main immune groups of TAMs, B cells, and T cells in glioma, and 352 TAM marker genes were obtained (Supplementary Table S1) and shown (Figure 3B).

### Identification of prognostic genes associated with M2 TAMs

**Figure 4:**
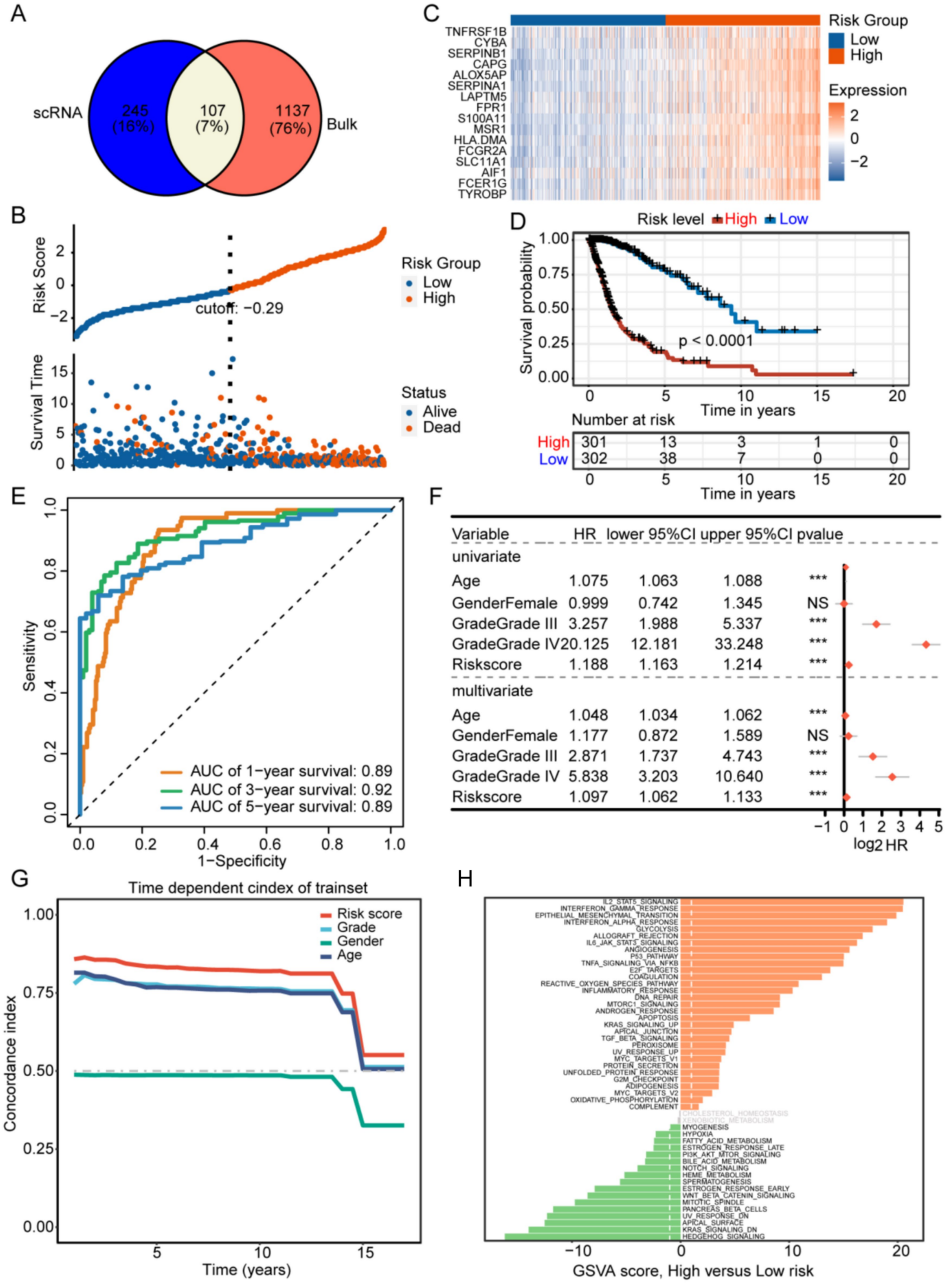
**(A)** Development of a prognostic signature associated with M2 tumor-associated macrophages (TAMs) identification of potential genes associated with M2-like TAMs. **(B)** The training set. **(C)** The prognostic gene expressions are presented for different groups. **(D)** The high-risk group in the training set showed a significantly worse prognosis according to Kaplan–Meier survival curves. **(E)** The prognostic efficacy of the risk score was confirmed using the AUC of the time-dependent ROC curves. **(F,G)** The univariate and multivariate analyses, along with the C-index, demonstrated that the risk score was an independent risk factor affecting survival more significantly than other indicators. **(H)** Results of GSVA comparing high- and low-risk groups.

Three hundred and fifty-two TAM marker genes were intersected with 1,244 M2 macrophage module genes, and 107 M2 TAM related candidate genes were obtained (Figure 4A and Supplementary Table S2). The univariate Cox and LASSO regression analyses identified 16 prognostic signature genes, including *AIF1*, *ALOX5AP*, *CAPG*, *CYBA*, *FCER1G*, *FCGR2A*, *FPR1*, *HLA-DMA*, *LAPTM5*, *MSR1*, *S100A11*, *SERPINA1*, *SERPINB1*, *SLC11A1*, *TNFRSF1B*, and *TYROBP* (Supplementary Figure S3 and Supplementary Table S3). The existing body of research has validated the involvement of these 16 genes in various immunological processes and tumor progression, including immunomodulation and signal transduction (AIF1) (13), immune checkpoint regulation (TYROBP, FCER1G, FCGR2A) (14, 15), metabolic reprogramming (ALOX5AP, MSR1, SLC11A1) (16–18), protease homeostasis (SERPINB1, SERPINA1) (19, 20), cytoskeletal dynamics (CAPG) (21), microenvironment remodeling (S100A11) (22), antigen presentation and immune evasion (HLA-DMA, LAPTM5) (23, 24), and tumor inflammatory regulation (FPR1, TNFRSF1B, CYBA) (25–27). These genes are implicated in immune regulation and may play a critical role in shaping the M2 tumor-associated macrophage (TAM) phenotype, thereby facilitating tumor progression and malignancy. The ALOX5AP inhibitor zileuton has been shown to decrease tumor-associated macrophage (TAM) infiltration in murine models (28), while targeting TREM2 and TYROBP may reverse immunosuppression (29). These findings indicate that these genes represent promising candidates for targeted therapeutic interventions.

### Development of a prognostic model associated with M2 TAMs

Patients with GBM in the training set were categorized into low-and high-risk groups based on an optimal risk score cutoff of −0.29 (Figure 4B). Figure 4D illustrates that overall survival (OS) was extended in the low-risk group compared to the high-risk group. The high-risk group exhibited elevated expression of 16 prognostic signature genes, indicating poorer prognosis (Figure 4C). The AUCs for predicting overall survival were 0.89 at 1 year, 0.92 at 3 years, and 0.89 at 5 years (Figure 4E). Univariate and multivariate analyses, along with the concordance index, demonstrated that age, grade, and risk score independently influenced survival (Figures 4F,G). The high-risk group showed enrichment of IL2-STAT5 signaling, interferon-gamma response, epithelial-mesenchymal transition, P53 pathway, and angiogenesis, whereas the low-risk group exhibited significant enrichment in hedgehog signaling, Kras signaling, heme metabolism, and fatty acid metabolism (Figure 4H).

### Validation of the prognostic signature associated with M2-like TAMs

**Figure 5:**
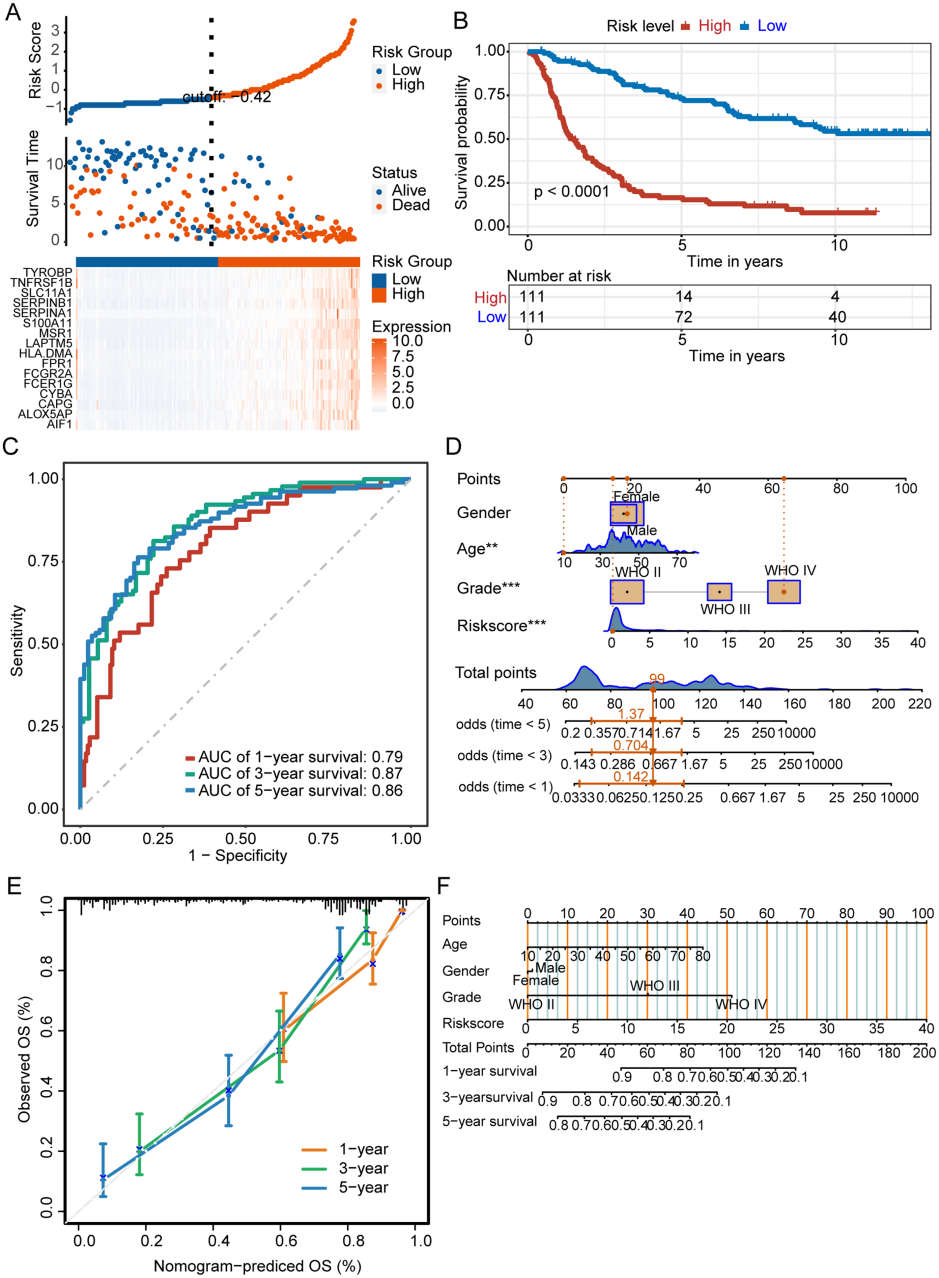
Prognostic signature for M2 TAMs. **(A)** The test set displayed risk scores, survival status, and prognostic gene expression for patients with glioma, categorized into different groups. **(B)** Kaplan–Meier survival curves of different risk groups. **(C)** ROC curves. **(D)** Nomogram integrating risk scores and clinical indicators. ^***^p < 0.001. **(E)** The nomogram demonstrated reliable performance, as indicated by the calibration curve.

We next validated the prognostic signature in the test set to confirm its reliability. Patients with GBM were divided into low-and high-risk groups using a risk score cutoff of −0.42 in the test set, with the high-risk group exhibiting elevated expression of 16 prognostic signature genes (Figure 5A). The prognosis of high-risk group was worse than that of low-risk group (Figure 5B). In the test set, AUC values predicting 1-year, 3-year, and 5-year overall survival were 0.79, 0.87, and 0.86, respectively (Figure 5C). The constructed M2-like TAM prognostic signature reliably predicts GBM patient outcomes. We developed a nomogram incorporating prognostic risk scores and clinicopathological indicators to comprehensively predict survival (Figure 5D). The reliability of the nomogram was verified by its calibration curve (Figure 5E).

### Immune cells related to risk signature and the immunotherapy landscape

**Figure 6:**
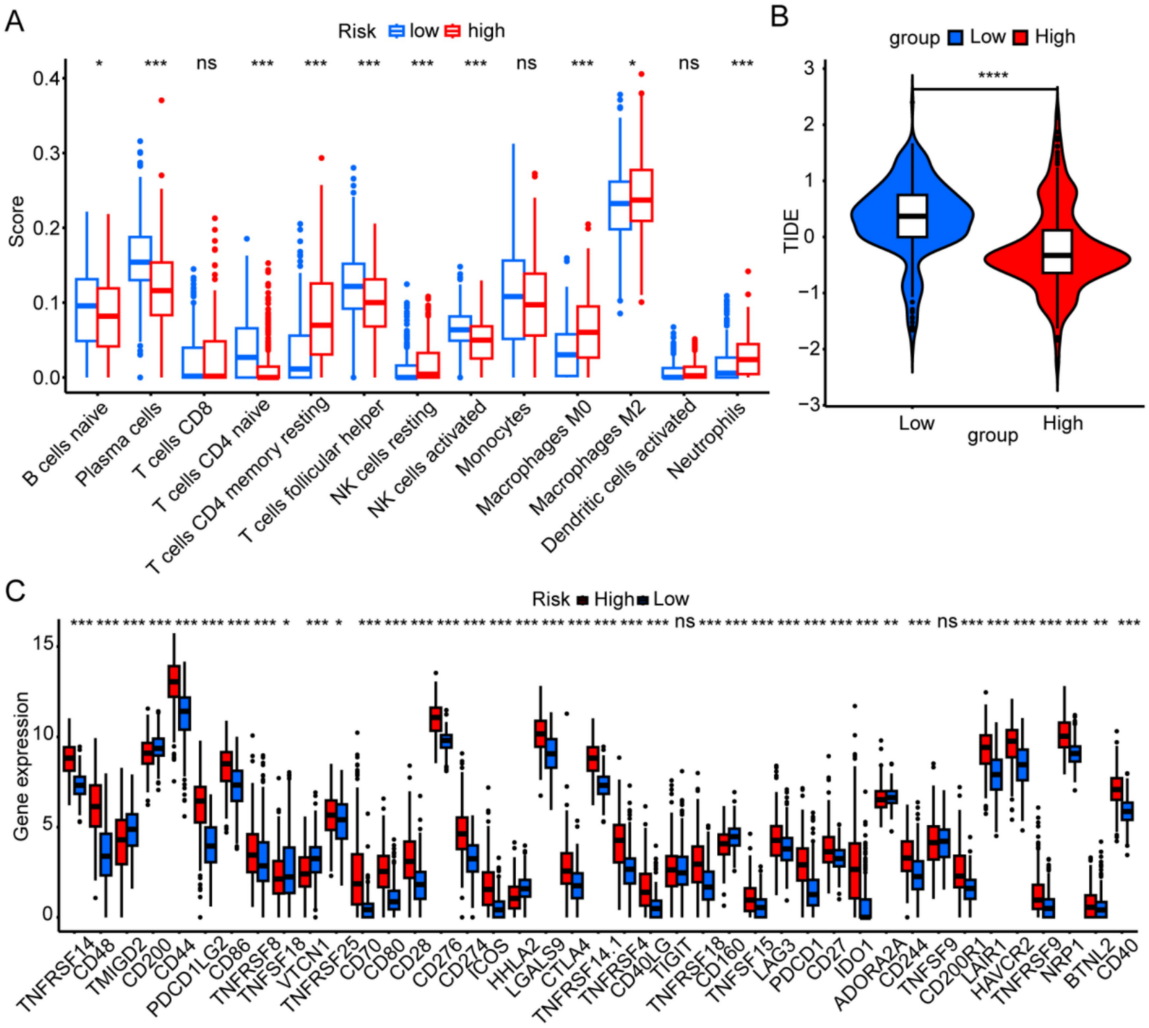
Immune landscapes associated with the six risk signatures. **(A)** Immune cell scores were analyzed to compare high- and low-risk groups. **(B)** The tumor immune dysfunction and exclusion scores were analyzed to compare high- and low-risk groups. **(C)** Immune-checkpoint gene expression was analyzed between different risk groups. ^*^p < 0.05, ^**^p < 0.01, ^***^p < 0.001, and ns, not significant.

In the high-risk group, B cells and activated NK cells were less abundant, whereas resting CD4 memory T cells and neutrophils were more prevalent (Figure 6A). Figure 6B shows that TIDE scores in the low-risk group were higher than those in the high-risk group, indicating that patients in the low-risk group were more prone to immune evasion and weakened response to immunotherapy. Our analysis of immune checkpoint-related gene expression revealed that several differentially expressed genes, including *TNFRSF14*, *CD44*, *PDCD1LG2*, *CD86*, *CD80*, and *CD276*, were significantly upregulated in the high-risk group, which may provide new targets for immunotherapy (Figure 6C).

### Tissue expression of signature genes

**Figure 7:**
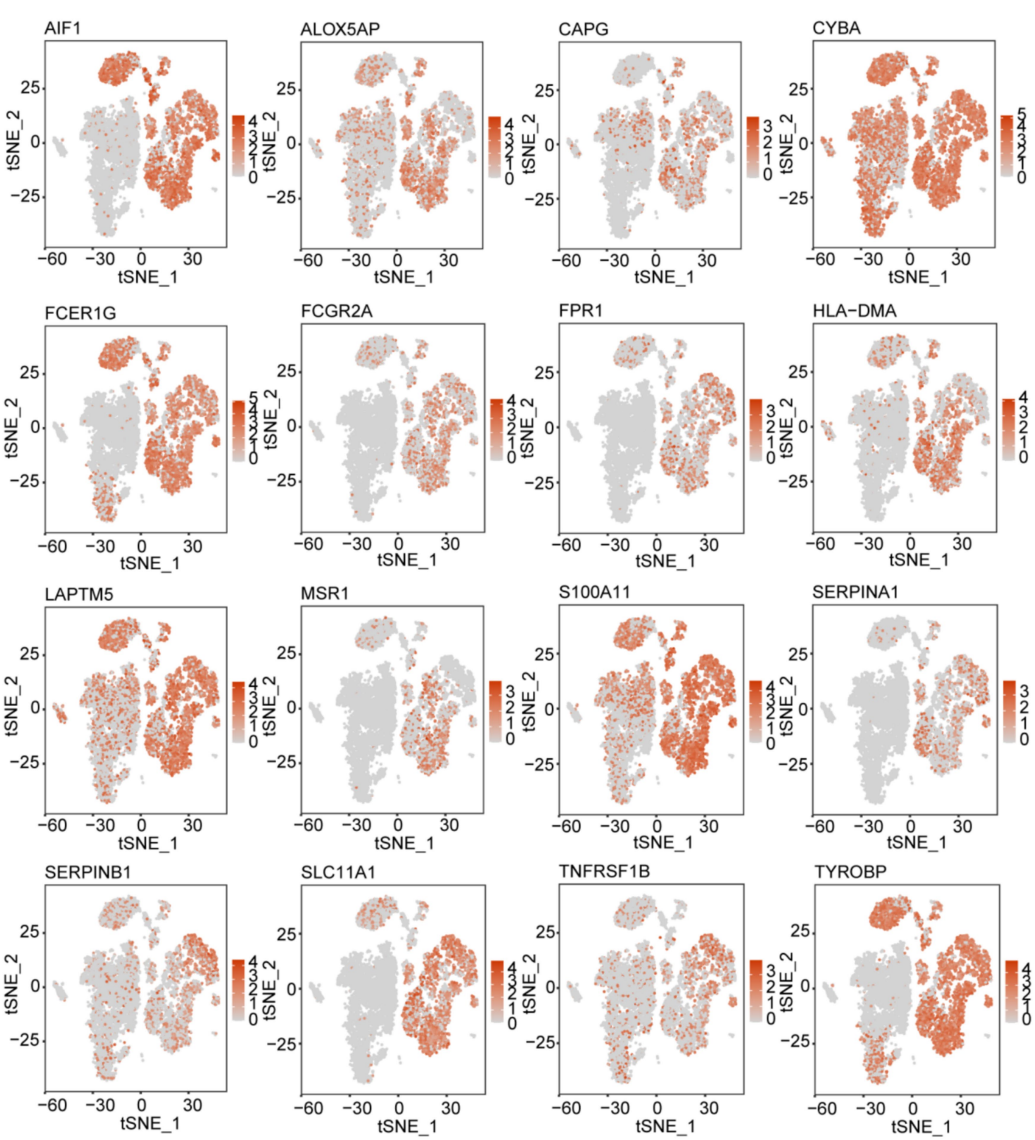
t-SNE map illustrating the expression patterns of 16 prognostic signature genes associated with M2-like TAMs.

**Figure 8:**
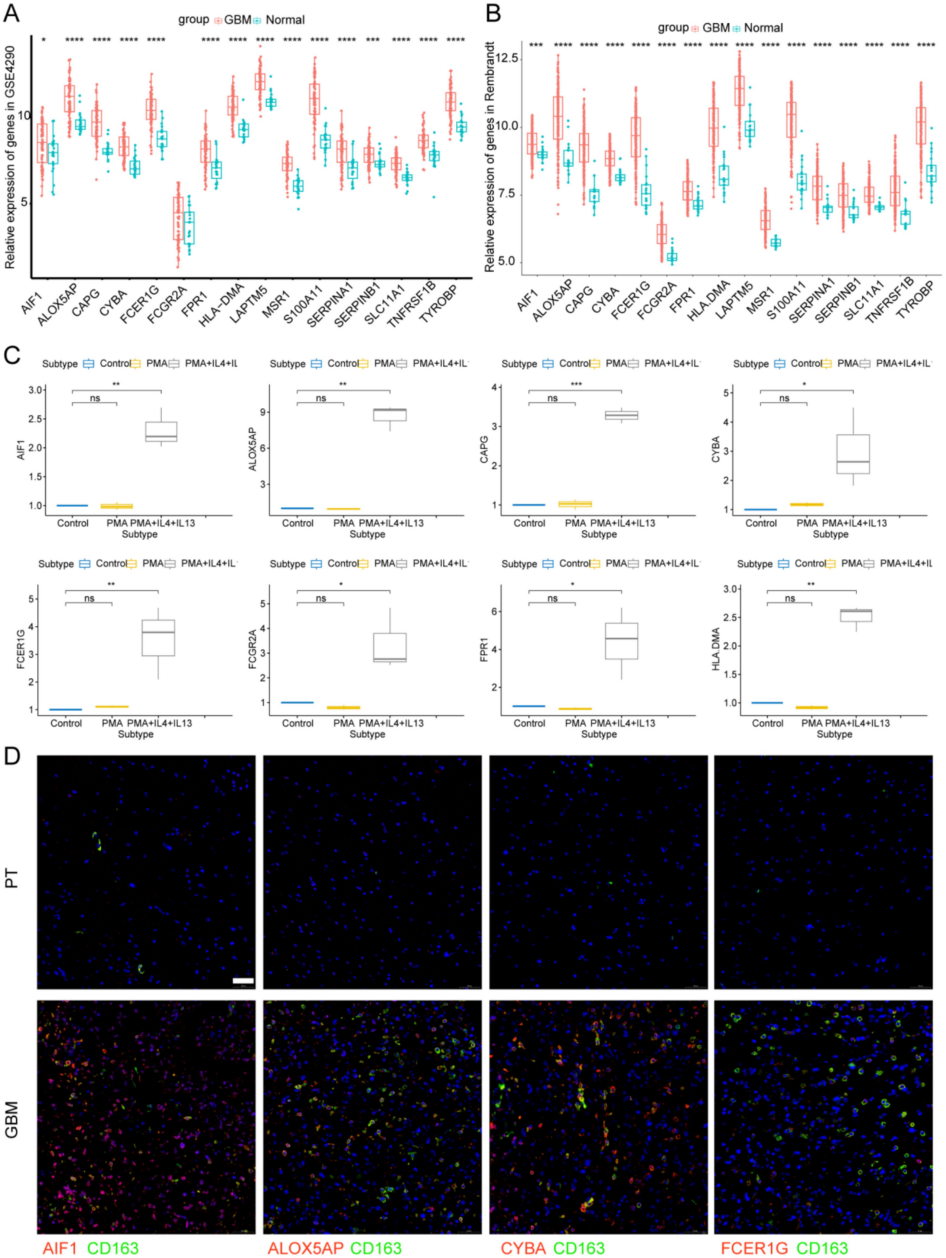
Signature genes expression levels in GBM tissues and M2 macrophages. **(A)** Signature genes expression levels in GSE4290 database and **(B)** Rembrandt database. **(C)** Signature genes expression levels were significantly upregulated in THP-1-derived M2 macrophages. ^*^p < 0.05 and ^**^p < 0.01. **(D)** Immunofluorescence images of signature genes (Red) and CD163 (Green) in GBM tissue. PT, para-carcinoma tissue. The scale bar in the column represents 50 μm.

We analyzed the signature gene expression using both single-cell and RNA-seq data from public databases. The results of scRNA-seq analysis showed that 16 signature genes were abundantly expressed in TAMs, and that *AIF1*, *ALOX5AP*, *CAPG*, *CYBA*, *FCER1G*, *FCGR2A*, *FPR1*, *HLA-DMA*, *LAPTM5*, *MSR1*, *S100A11*, *SERPINA1*, *SERPINB1*, *SLC11A1*, *TNFRSF1B*, and *TYROBP* were relatively specifically expressed in TAMs (Figure 7). The expression levels of the 16 signature genes in GBM samples were significantly upregulated in the GSE4290, Rembrandt databases and THP-1-derived M2 macrophages (Figures 8A–C; Supplementary Figure S4A). In addition, these genes are highly expressed in M2 macrophages in human glioblastoma tumor tissues (Figure 8D; Supplementary Figure S4B).

## Discussion

The immunosuppressive glioma TME significantly contributes to GBM progression (30). The intricate interactions between tumor cells and the glioma TME contribute to GBM progression and resistance to therapy. M2 macrophages are the main immune cells in glioma TME, and play an important role in glioma angiogenesis, invasion and immune escape (31). Investigating the influence of M2 TAMs on glioma progression and creating a prognostic signature linked to M2 TAMs are crucial. In this study, we identified 16 prognostic genes related to M2 TAMs and consequently, developed and confirmed a prognostic signature for predicting GBM outcomes.

Our findings indicate that patients with glioma with elevated immune scores or microenvironmental content have poorer prognoses, linking the microenvironment to glioma outcomes. Patients with glioma with elevated levels of macrophages or M2 macrophages exhibited poorer prognoses, indicating the significant role of M2 macrophages in glioma outcomes. By crossing 352 TAM marker genes with 1,244 M2 macrophage module genes, a total of 107 candidate M2-TAM genes were identified. Sixteen prognostic signature genes (*AIF1*, *ALOX5AP*, *CAPG*, *CYBA*, *FCER1G*, *FCGR2A*, *FPR1*, *HLA-DMA*, *LAPTM5*, *MSR1*, *S100A11*, *SERPINA1*, *SERPINB1*, *SLC11A1*, *TNFRSF1B*, and *TYROBP*) were identified for the construction of a prognostic signature for glioma. Several characteristic genes have been found to be associated with glioma prognosis. Activated macrophages and microglial cells expressing AIF-1 have been reported to be linked to tumor malignancy and poorer prognosis (32). Moreover, *FCER1G* has been shown to be significantly elevated in high-grade glioma and predicted a poor prognosis (33). Furthermore, increased *CAPG* expression is associated with enhanced immune cell infiltration and poor survival of glioma (21). A previous study demonstrated that *MSR1* inhibition suppressed migration, invasion, epithelial-mesenchymal transition, and proliferation in lower-grade glioma (34).

In the present study, patients with GBM in the training set were categorized into low-and high-risk groups. The high-risk group exhibited a notably poorer prognosis than the low-risk group. The prognostic signature was validated on the CGGA325 dataset, confirming its applicability and validity. Our signature independently predicted GBM prognosis in the training set, as evidenced by the ROC curves, *C*-index, and the results of univariate and multivariate analyses. We also constructed a nomogram to confirm that risk scores associated with the prognostic signature independently predicted prognosis in the test set. GSVA showed that metabolism-related pathways were more abundant in the low-risk group, while tumor-related pathways including epithelal-mesenchymal transformation and p53 pathway were significantly enriched in the high-risk group, which may explain the poor prognosis in the high-risk group.

Prior research has focused on the connection between prognosis and immune cells in the TME for many cancer types. Tumor cells inhibit the antitumor immune response by attracting or expressing immune checkpoints (35). The prognostic signature in the risk group was linked to the immune landscape and response to immunotherapy. The high-risk group exhibited a decrease in B cells, Th cells, and activated NK cells, whereas resting CD4 memory T cells and neutrophils were elevated. The high proportion of B cells suggested a better prognosis (36). Reduced NK cell activity has been reported to facilitate tumor immune evasion and correlate with poorer prognosis (37). Moreover, unbalanced T1/T2 expression causes immune escape and contributes to tumor progression in bladder cancer (38). Additionally, intratumor neutrophils are an independent adverse prognostic factor, and their reduction is associated with shorter survival in patients with colorectal cancer (39).

We analyzed the expression of prognostic signature genes in single-cell and bulk RNA-seq databases and found that all 16 signature genes were abundantly expressed in TAMs. Prognostic characteristics have the potential to inform immunotherapeutic strategies by predicting the immune landscape. Patients identified as high-risk, characterized by markers such as FCER1G and TYROBP, display “cold tumor” phenotypes, typified by low infiltration of CD8 T cells and elevated levels of PD-L1-expressing macrophages. This profile suggests a likely resistance to anti-PD1 therapies but indicates a potential responsiveness to CSF1R inhibitors. Furthermore, these high-risk patients might derive additional therapeutic benefit from adjuvant treatments, such as the ALOX5AP inhibitor zileuton.

Our study was subject to several limitations. Publicly available datasets frequently lack comprehensive treatment histories and detailed clinical data, which can introduce confounding factors in survival analyses. Future studies may investigate functional assays conducted in laboratory settings and *in vivo* to make a more profound understanding of the mechanisms by which macrophage-associated signature genes influence glioblastoma (GBM). For instance, macrophages could be co-cultured with glioblastoma cell lines after knockout of signature genes (e.g., FCGR2A) to evaluate M2 polarization and observe changes in tumor cell biology (40). Additionally, inhibitors targeting signature genes identified in this study [e.g., the ALOX5AP inhibitor AM679 (41)] could be administered to mouse glioma orthotopic models (e.g., GL261) to assess tumor growth dynamics and immune microenvironment remodeling. These approaches may inform future research directions aimed at exploring the effects of signature genes on M2 macrophages within the glioma microenvironment.

## Conclusion

In this study, we developed a prognostic signature associated with M2 TAMs to predict patient outcomes in glioma. The prognostic signature provides potential targets for glioma therapy.

## Data Availability

Publicly available datasets were analyzed in this study. This data can be found here: GSE147275: https://www.ncbi.nlm.nih.gov/geo/query/acc.cgi?acc=GSE147275, GSE4290: https://www.ncbi.nlm.nih.gov/geo/query/acc.cgi?acc=GSE4290, Rembrandt: http://gliovis.bioinfo.cnio.es, CGGA325: http://www.cgga.org.cn/.

## Footnotes

^1^ http://gliovis.bioinfo.cnio.es/

^2^ http://www.cgga.org.cn/

## Supplementary material

SUPPLEMENTARY FIGURE S1 Relationship between immune cells and prognosis.

SUPPLEMENTARY FIGURE S2 Preprocessing and analysis of single-cell RNA-seq data.

SUPPLEMENTARY FIGURE S3 Identification of the prognostic signature in the training set.

SUPPLEMENTARY FIGURE S4 **(A)** Signature gene expression levels in THP-1-derived M2 macrophages. ^*^*p* < 0.05 and ^**^*p* < 0.01. **(B)** Immunofluorescence images of signature genes (Red) and CD163 (Green). The scale bar in the column represents 50 μm.

SUPPLEMENTARY TABLE S1 List of 352 TAM marker genes identified via scRNA-seq analysis.

SUPPLEMENTARY TABLE S2 List of 107 intersecting genes.

SUPPLEMENTARY TABLE S3 Results of univariate Cox regression and LASSO regression analyses.

SUPPLEMENTARY TABLE S4 Quantitative real-time PCR primers of signature genes.

SUPPLEMENTARY FILE S1 R working documents.
