# Supplementary figures and images for: Biomarker identification associated with M2 tumor-associated macrophage infiltration in glioblastoma
Source: Front Neurol. 2025 May 14;16:1545608. doi: 10.3389/fneur.2025.1545608 (PMC12117037; doi:10.3389/fneur.2025.1545608)

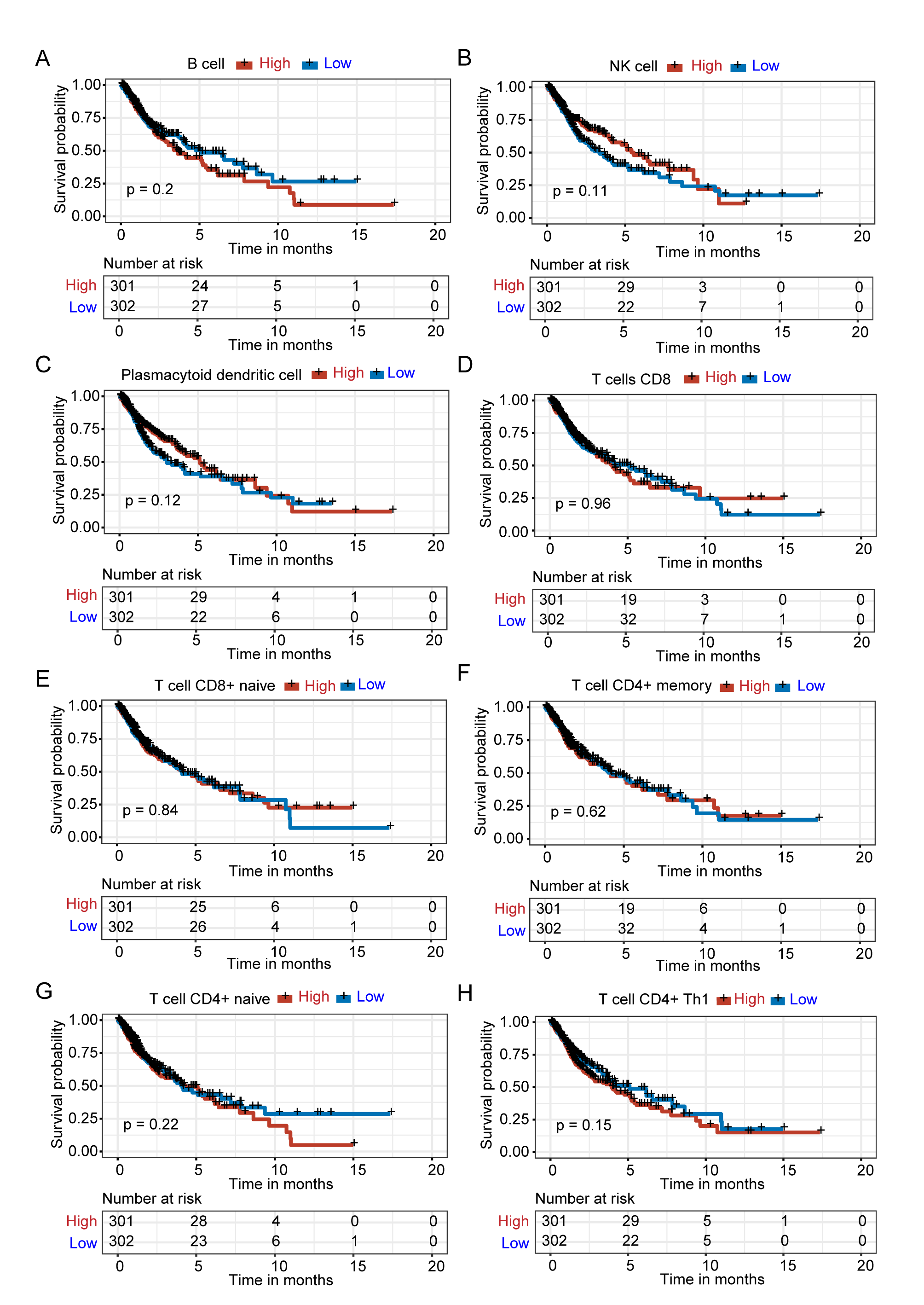

Supplement: SUPPLEMENTARY FIGURE S1 — Relationship between immune cells and prognosis. [file Image_1.tif]

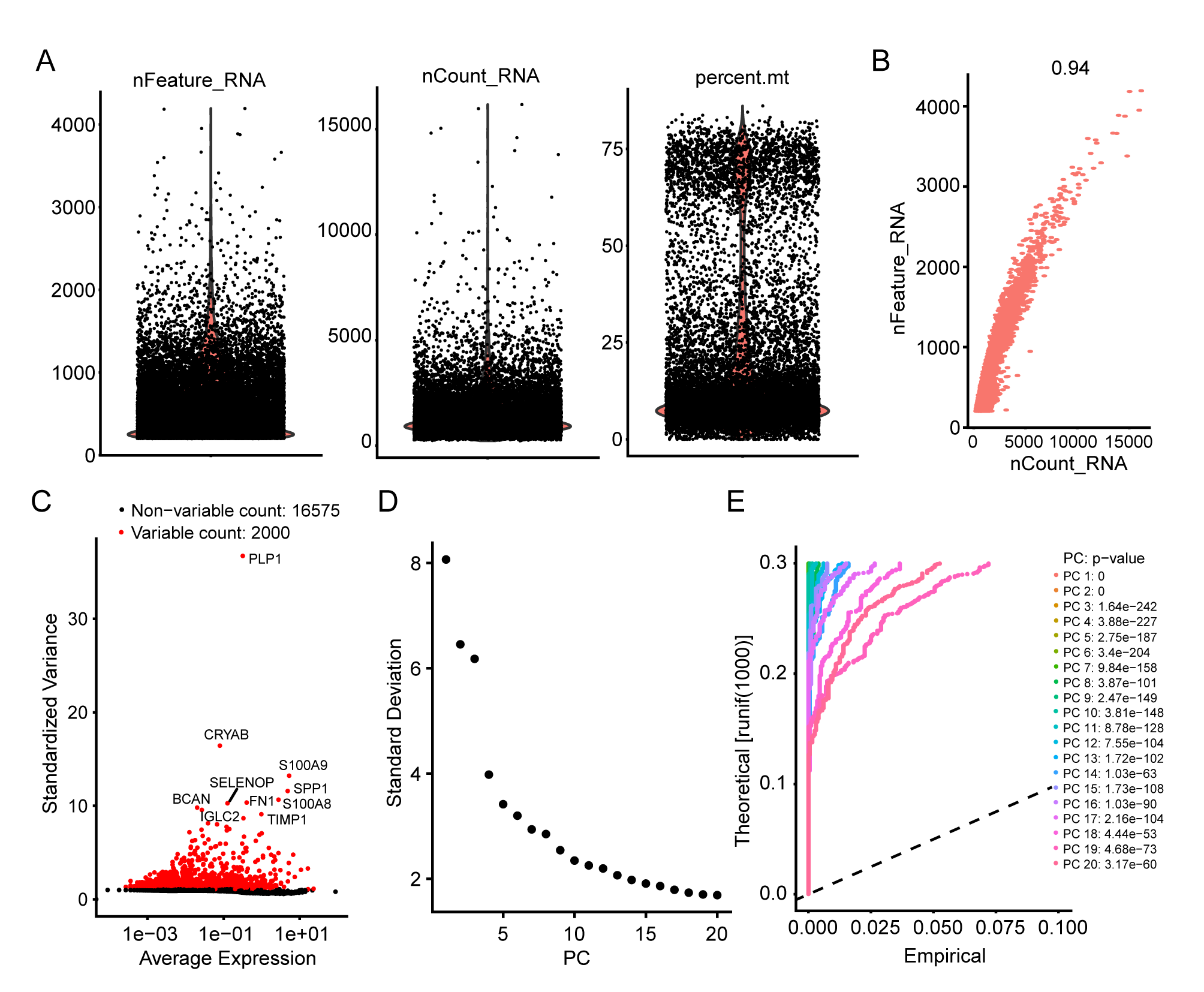

Supplement: SUPPLEMENTARY FIGURE S2 — Preprocessing and analysis of single-cell RNA-seq data. [file Image_2.tif]

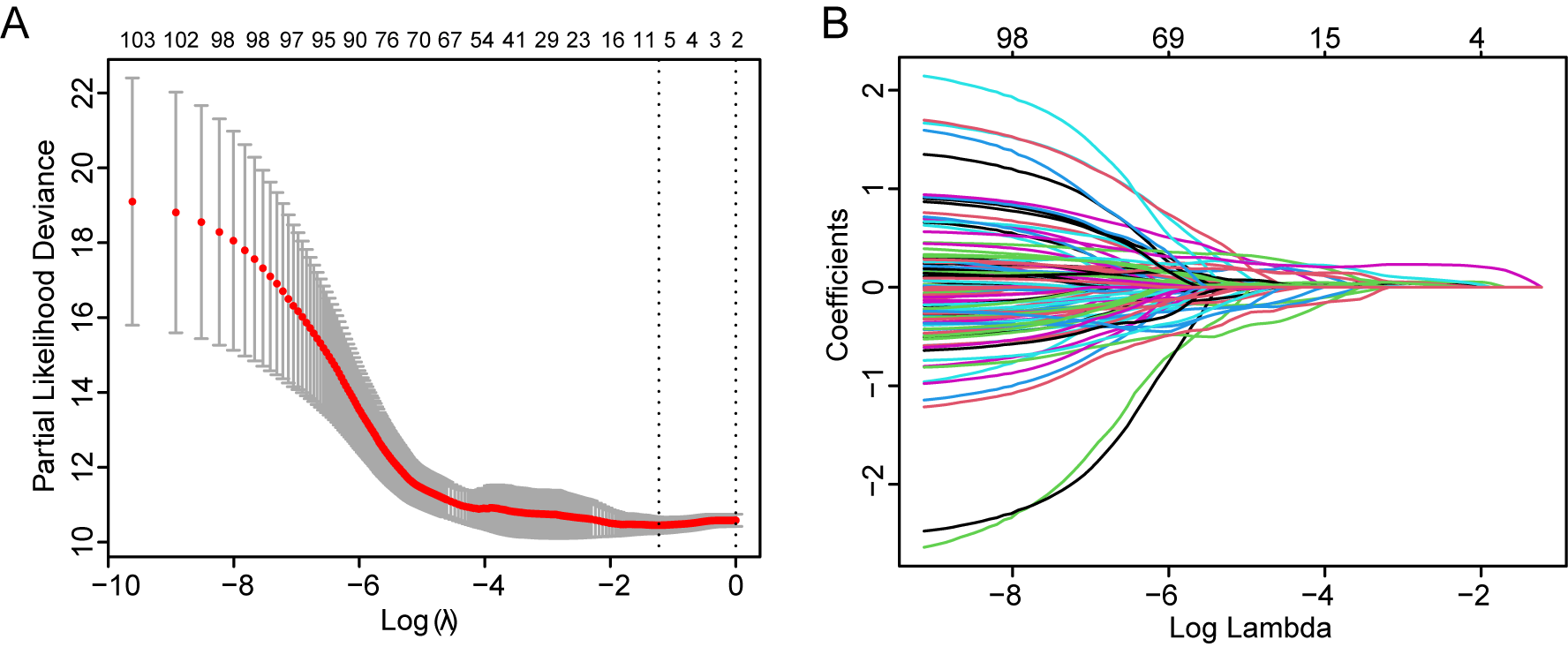

Supplement: SUPPLEMENTARY FIGURE S3 — Identification of the prognostic signature in the training set. [file Image_3.tif]

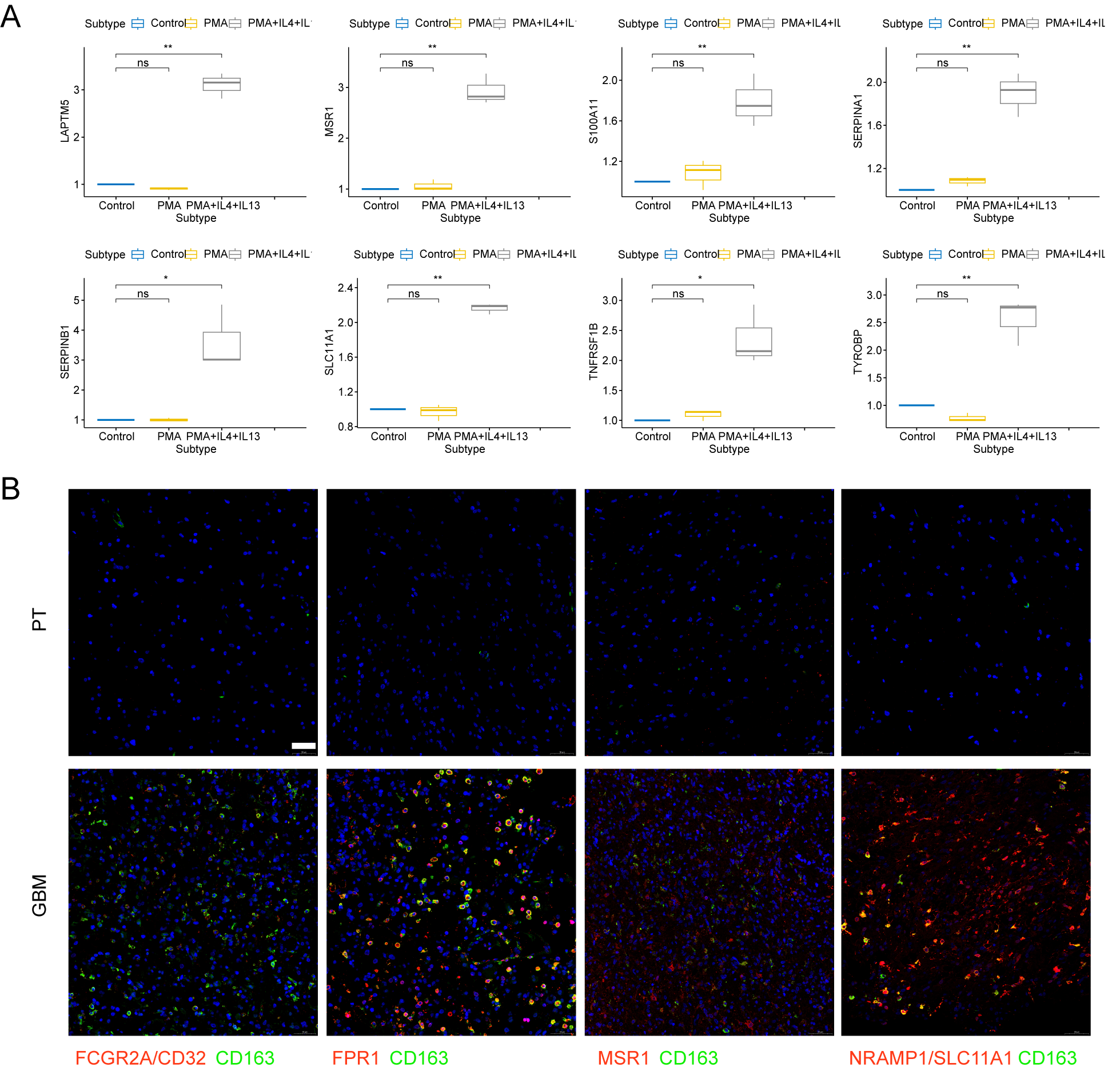

Supplement: SUPPLEMENTARY FIGURE S4 — (A) Signature gene expression levels in THP-1-derived M2 macrophages. *p < 0.05 and **p < 0.01. (B) Immunofluorescence images of signature genes (Red) and CD163 (Green). The scale bar in the column represents 50 μm. [file Image_4.tif]
